# To What Extent and When Are We Justified in Using Cataphoresis? Is There Danger of Injuring the Dental Pulp or Other Tissues, by Its Use

**Published:** 1897-09

**Authors:** H. T. Harvey

**Affiliations:** Battle Creek


### To What Extent and When are We Justified in Using Cataphoresis? Is There Danger of Injuring the Dental Pulp or Other Tissues by its use.

BY DR. H. T. HARVEY, OF BATTLE CREEK.

Read before the Michigan State Dental Society.

The history of the discovery of the principal underlying the cataphoric method presents some features of more than ordinary interest.

Especially does it show that great study, together with constant effort is required, and then only by slow progress are tangible results obtained. As has been said, "No great truth springs into completeness from one thought in an instant, but rather by an evolutional process, in which its possibilities are gradually developed and utilized for the benefit of humanity," and from that ever-flowing spring of thought and genius has been handed down to us the great principal of methodical cataphoresis.

It is by no means new. Members of the medical profession have experimented with electro-chemical anæsthesia as early as

1833, and quite extensive experiments were performed both in medical and dental surgery in 1859.

The results of experiments at that time were defined as follows: voltiac narcotism was attended with some eminent successes, and also with some singular failures, and that all objections to this method were removable.

It seems that the essential features of cataphoresis, after remaining in a dormant state for half a century or more, is resurrected, placed in a different aspect, or new light, and its practical utility brought forth.

The principal factors responsible for the resuscitation of cataphoresis is the intense interest manifested in utilization of electricity in dentistry. The therapeutic and analgesic properties of cocaine salts, and the constant demand for the alleviation of pain caused our patients in the more severe operations in dental surgery.

It is not my intention, at this time, to discuss the ancient basic features of cataphoresis or detail its recent origin. This has been very ably placed before us, and I will now devote my time to cataphoresis as we find it to-day.

We who are interested in cataphoresis have now had the practical experience for a year or more of its use in our several practices, and no doubt, all acquiesce, though our experience has been limited, we have used it sufficiently to admit of some merit in the principal involved in its use.

There can be no doubt however, but that there is room for improvement in devices for managing the electric current, and a decided need of an extended investigation in producing more effective medicinal solutions suitable for therapeutic treatment. It is not by any means conceded or demonstrated that cocaine is the best drug to use as a dental obtundent, and there is much reason to believe that there is great merit in other drugs if developed.

Promoters of medical and dental science fifty years ago, with the salts of cocaine, unknown, produced wonderful results, and yet they were confronted with one of the great obstacles that we ourselves are unable to meet, that is imperfect electrical devices, and by that I mean that the problem of a continuous current, where by it remains unbroken from first application until complete anæsthesia is produced.

The whole field of dental applications of the cataphoric principal is still in its infancy; diligent search for a complete realization of all its possibilities is required.

To what extent, and when are we justified in using cataphoresis? Surely you will agree with me, when I say that not all patients we operate upon demand dental obtundents.

The question is, what class of patients demand cataphoric applications? To what extent should we go with those classes? How shall we differentiate between them?

A thorough study of temperaments is imperative in treating patients with cataphoresis; we should also understand thoroughly the maximum and minimum amount of electricity each case requires, and will tolerate, and yet, produce thereby the desired anæsthetic effect.

We should know what medicinal solutions are indicated in each case, where application is necessary. There are principals to be followed; positive rules to be established, studied and observed in order to co-operate with all other advancing, improved methods and devices, thereby rendering a perfectly successful operation possible.

While in attendance at the annual session of the American Dental Association at Saratoga, last August, I was greatly interested in hearing related some experiments which an inventor of an electrical dental apparatus, and an expert of a New York life insurance company had made, testing the capacity of different individuals as to the degree and amount of electricity they would tolerate, and what was requisite to produce contraction of various muscles. Brothers would vary from twenty to thirty-five volts. Father and daughter varying the same degree. Mother and son would require each an equal amount. The results of their experiments forced them to the conclusion that only by various tables of temperaments, with general diagnostic features could a safe and reliable rule be adopted.

Another important and great obstacle is, time required in some cases, although I believe a skillful operator can, to some extent overcome this objection.

When are we justified in using a dental obtundent for excavating dental decay? I must define that question by my experience. I ascertain from a temperamental knowledge to some degree, what temperament the patient I am to operate upon possesses. If undecided by a causual glance, I test sensitivity of tooth with my excavator. If I find hypersensitive condition I make cataphoric applications in the usual manner. From records made in my practice in the past six months I was required to use an obtundent in 250 percent of the cases operated upon; out of one-quarter of the cases operated upon, in which cataphoresis was used, twenty-seven percent of those cases were absolutely successful, in seventy-three percent of the cases, pain from mild to intense, followed the application of electricity. I also found that all cases that proved satisfactory were of sanguine or sango-billio temperament, and the patients who complained of intense and at times intolerable pain accompanying the application of the electrical current, the minutest fraction of a volt, were those dark-haired, black-eyed, nervo-bilious-tempered individuals. They possess teeth so sensitive that they respond to the slightest fluctuation of the current. I believe that could we produce a non-fluctuating, non-breaking current that by gentle, continuous applications of electricity, the most pronounced type of hypersensitive teeth could be reduced to perfect anæsthesia. With all other classes I have no trouble. There is in some cases that disagreeable sensation, as some choose to term it, upon application, and upon each succeeding increase of voltage of the current.

What current and to what extent should we use cataphoresis, must also be regulated by one's experience in cataphoric operations. To my mind the only safe and satisfactory one is a primary and secondary current; a commercial current is always unsafe, danger from cross wires; and is very unsteady, and I know of many prominent dentists who used a commercial current for their cataphoric operations, and found the current too unsteady, and also unsafe, and have resorted to the use of the battery.

To what extent we should use this method, each must determine. Should every case indicate the application of cataphoresis, just to that extent should he use it. As to voltage required to produce structural anæsthesia, it usually requires from twenty-five to forty volts, no more should be applied, and in a majority of cases twenty-five volts will suffice. Fifteen volts is the minimum amount. I have been able to produce total anæsthesia. From ten to thirty minutes are required usually to anaesthize a tooth.

I have also used Prof. Morton's Electrode No. 1 in lancing alveolar abscesses and also his No. 2 Electrode (deepest) in extraction with excellent success.

Is there danger of injuring the dental pulp or other tissues by the use of cataphoresis? I find by investigation that about ninety-five percent of the cases where the cataphoric application is made, that the tooth structure is much more sensitive than before, and where metallic fillings are placed in immediate contact with the structural walls of the cavity, there is greater danger from internal shocks to the pulp than there would be ordinarily where there is no cataphoric application made.

I think that with an intermediate non-conducting lining in all cavities treated by this method the danger from this source would be reduced to a minimum. I invariably follow the practice as outlined. I have learned of only one devitalized pulp resulting directly from the electrical current, and that to my mind may have been diseased, and the current or volt of the current indiscriminately used.

There is reported a case of toxemia or toxicosis by Dr. Henry I. Moore, of Frankfort, Germany, during an operation of pulp extripation and produced toxicosis by a surplus amount of cocaine, it however serves to illustrate the power of cocaine upon the more delicate tissues, and why not upon the structure of a tooth.

I have been unable to discover any serious after-effects from the use of cataphoresis where the necessary precautions have been observed, as outlined above.

And could we apply the electric current without producing those painful sensations I would think we had reached the mecca in dentistry, therefore, as I have mentioned before, the results thus far attained, while encouraging are doubtless capable of improvement. I use Wheeler's Fractional Volt Selector, with thirty-cell battery and guiaca cocaine as a medicinal solution.

DISCUSSION.

Dr. Morse: I had a cataphoric apparatus at very early period of its use in its present condition. I have been troubled in obtaining a desirable current throughout my entire experience, and the result is that I have not had as much service from it as many of you. I attempted to use a storage battery of ten cells with light plates and for a time they were very successful, but became short-circuited and were soon exhausted. I then attempted to use primary battery cells in connection with a volt selector made for 110-volt circuit and with that I have not had the success I should have had. The success I have had in some cases has been very pleasing and in other cases I have had to spend so much time that I have thought that we were hardly justified in the result gained, considering the time expended unless very well remunerated. Hyper-sensitive dentine is certainly the condition indicated for the use of cataphoresis. There are many cases, as we all know, where it is not necessary to use an obtundent. In a talk I had with Dr. Custer at Grand Rapids last year, he related some of his experiences. His theory of the reason why we did not get quicker and better Cataphoric action in the use of cocaine by the aid of electricity, coincided fully with my own observations. The tubuli extend from the pulp in an outward direction and they end in the cavity; uncovered they will be the best conductors of the electrical current, and will carry the medicine that is placed in the cavity through the tubuli to the pulp. The tubuli that extend into the dentine, about the cavity, and whose outer ends were covered will not receive and conduct the current in the same manner. The drug will be carried to the pulp through the open tubuli and the pulp will be anaestheized at the point where the tubuli entered the pulp chamber and it would be necessary to anaesthetize that pulp sufficiently so that the tubuli connected with the pulp and extending through the dentine around the cavity would be anaesthetized before we could make lateral excavations or under-cuts, or drill in that direction. I think this is the reason it requires so long to get anaesthesia in certain cavities, particularly in those portions where we wish to obtain under-cuts.

Question: About how long a time does it generally take to obtain anaesthesis?

Dr. Morse: It varies a great deal. I have had satisfactory results in twelve minutes and even shorter time than that; and again in twenty minutes I have had no satisfactory results; but after the first application by removing the intervening decayed dentine, I think it hastens the operation and I have had better results in making two or three applications, in making partial excavations and then re-applying. In regard to being unable to determine the amount of current to be used upon a patient I have not been able to find any method by which this could be done. From my own observations and reading I am led to believe that it is impossible to determine by any outward indication the length of time, the amount of current, or the strength of the solution necessary to produce anaesthesia.

Dr. Blackmarr: I am surprised that Dr. Harvey is not as enthusiastic over this subject as he was last year. In the use of cataphoresis it seems to me, it is like everything else we use; it requires judgment and tact. It reminds me of the saying of an eccentric uncle of mine, "It beats all how many times a day you can use good, common sense." The most success I have had was with exposed pulps. I have had success in obtunding decayed dentine and irritable dentine. I have a case in mind, shortly after I got the apparatus, a young lady who broke off three incisors, exposing the pulps. I applied cocaine crystals in solution to have it as strong as possible and in fifteen minutes I had the first pulp removed, perfectly painless. I took the three out in forty-five minutes.

Question: How long do you think it would have taken without cataphoresis?

Dr. Blackmarr: I don't know, but I am sure I could not have done it without the apparatus. She was a very sensitive patient.

Dr. Hall: I recently saw a patient immediately after the use of the cataphoric appliance on a lower bicuspid, the lip on the inside was black, and a spot on the outside of the cheek looked as though it was pretty well blistered. I would like some of you to tell me the cause.

Dr. Siddall: How much current was used?

Dr. Hall: On the first application the negative pole was applied to the cheek underneath the rubber dam. The twelve cells were used and in about twenty minutes the patient said she felt no effect of the current at all. The twelve cells were turned on again and the resistence used. The tooth being still sensitive, the patient felt no inconvenience; then all the resistance was removed and she said there was a sort of tingling sensation on the cheek, not enough so that she felt alarmed, and did not know the effect until after the apparatus was removed.

Dr. Siddall: I think it was the current. I have made experiments upon myself and have never been able to use more than six or seven wet cells, and eight dry ones was the limit. I am using the thing to avoid pain.

Dr. Fritz: I may be a little out of order, but I believe Dr. Blackmarr could have operated on those three pulps in less time with equally good results with a two or three percent solution of cocaine. I had, about a year ago, a similar experience, not quite so favorable. I don't think I took over fifteen minutes for the operation and it was painless.

Dr. Loeffler: We want to know the actual result from the application of cataphoresis, much depends upon the patient. I remember a case that was brought up at our local association by one who has had, in a measure at least, wonderful success with the apparatus. He had made every effort to make a painless operation and after tiring himself out for half the afternoon, and the patient showing no indication of any insensibility, to his utter disgust upon asking him how he liked the operation, the patient replied, "It hurts like the mischief." It seems a great deal of the result we get, depends upon the veracity of the patient.

Dr. Raymond: I am surprised that Dr. Harvey did not show more enthusiasm in regard to cataphoresis and that he has had so large a percentage of failures. One year ago I commenced using Dr. Custer's apparatus. I have used it quite a good deal since and my percentage of failures has been much less than Dr. Harvey's. I am surprised at Dr. Blackmarr's statement, that Dr. Custer said that in five years' time he did not suppose there would be one of his appliances in use. It is different from his statement to me last summer. He said he did not think you could take a case to him he could not be successful with. Dr. Harvey said the minimum amount of voltage was fifteen. It may be so with the Wheeler. I had forty used on a tooth of mine and stood it very well. I use Dr. Custer's and you can not use more than twenty. You can use forty with success, but I have never gone beyond twenty. I have heard that those using the Wheeler have known their patients to jump a little as though from a break in the current. I don't know the difference in them. I have no doubt but that cataphoresis has come to stay and will be used by careful men who select their patients and know how to use it. If you have an inflamed pulp, it always causes pain as soon as the application is made. If you only have an exposure, you nearly always have success with it. You ought, if possible, to cut away the leathery portions of decay, before the application, as that is a non-conductor. Dr. Custer recommends cutting away the enamel so you can send the current through the tubuli, and it will take effect very much more quickly. I have used both cocaine and eucaine. A saturated solution of cocaine has given the most beneficial results.

Dr. Field: I use a Custer apparatus with a direct current. I think the current from a battery would be more satisfactory.

The first I ever used cataphoresis, an agent came to the office and wanted me to try his apparatus. I told him to take it up to the college as I would have a better chance for clinics there. He took it there and set it up. You want sensitive dentine or pulp if you are to be able to show anything. He brought the apparatus up there and I started in on the four percent solution. I tried that on one of the students. He said his teeth were extremely sensitive and had one of his teeth filled with gutta percha.

I had that removed. It was extremely sensitive. We used the three percent solution for about twenty minutes. We found part of the tooth obtunded and part of it not. I then tried four percent solution for thirty minutes and we could then cut as much dentine as we wanted to. I cut close on the pulp and there was no pain whatever, and he had never had any ill effect from it. I must say that my apparatus has been very satisfactory to me. I am surprised that a man like Prof. Taft will say that in five years' time it will not be used. I think ten will be used where one is now.

Dr. Blackmarr: That was Dr. Custer's statement to Prof. Taft. Probably a confidential one.

Dr. Harvey: I think one needs to use a good deal of hypnotism when applying cataphoresis. I think hypnotism has a good deal to do with the result.

